# Editorial: Steroid receptors in neuron and glia

**DOI:** 10.3389/fendo.2024.1472908

**Published:** 2024-08-14

**Authors:** Damien Le Menuet, Ioannis N. Charalampopoulos, Rebecca L. Cunningham, Konstantinos Kalafatakis, Ivan Nalvarte

**Affiliations:** ^1^ INSERM UMRS 1124 (T3S), Faculty of Basic and Biomedical Sciences, Université Paris Cité, Paris, France; ^2^ Division of Basic Sciences, School of Medicine, University of Crete, Heraklion, Greece; ^3^ Institute of Molecular Biology and Biotechnology (IMBB), Foundation for Research and Technology Hellas (FORTH), Crete, Greece; ^4^ Department of Pharmaceutical Sciences, System College of Pharmacy, University of North Texas Health Science Center, Fort Worth, TX, United States; ^5^ Barts and The London School of Medicine and Dentistry, Queen Mary University of London, London, United Kingdom; ^6^ School of Medicine, University of Crete, Heraklion, Greece; ^7^ Department of Neurobiology, Care Sciences and Society, Karolinska Institutet, Solna, Sweden

**Keywords:** steroid receptor, brain, glia, neurons, sex hormones, glucocorticoid

Steroid hormones comprising sex hormones and corticosteroid hormones (glucocorticoids and mineralocorticoids) act through members of the nuclear receptor superfamily of transcription factors but also through membrane receptors mediating fast cellular responses (1). These compounds regulate many functions in the peripheral and central nervous system such as mood, behavior, neurogenesis, myelination, and neuroprotection. The study of steroid actions on these processes are crucial to better understand neurological diseases (2). After decades of work, there are still many unanswered questions in this field of research. In the present Research Topic, we present original papers and reviews on the role of these receptors on neurons and glial cells that shed new lights on this field.

Glial role goes well beyond the support and protection of neural cells (3). Simões-Henriques et al. describe in an original study the influence of sex hormones on microglia morphology and the subsequent consequences on rodent anxiety behavior Early postnatal surge in testosterone in newborn rats is crucial for masculinization of the male brain and for male behavior (4). The masculinization of newborn rat females by testosterone results in an increased complexity of processes of microglial cells in adolescence and adulthood that is more similar to male microglia. This is associated with masculinization of the female behavior assessed by elevated plus maze and openfield anxiety behavior tests.

Membrane steroid receptors belong to various unrelated families of protein (5). They allow faster biological cascades than classical steroid receptors as the action mechanism does not necessitate the activation or repression of target genes. In an original rese arch Bradshaw et al. reveal sex differences in the expression of the membrane androgen receptor in different hippocampal regions and in the striatum of young adult rats. Further, they found that brain AR45 expression was unaffected by hypoxic exposure. These findings may open new avenues in the understanding of sex differences in neurological disease susceptibility.

Steroid receptors are present in all vertebrates including fishes (6). Boueid et al. reviewed the state of the art concerning the study of estrogen receptor roles in neural development using the zebrafish as a model This organism in which genetic modifications strategies are well established present an external embryonic development facilitating neurodevelopmental studies. Although it is well established that sex hormones are important players in neurodevelopment (7), the cellular and temporal contribution of the different estrogen receptors (ERs) during neurodevelopment is less well understood. To this end, Boueid et al. highlight findings on ER signaling during neurodevelopment in zebrafish and compare these findings to rodent data, illustrating the zebrafish embryo as an amenable *in vivo* model of ER signaling studies in both on the central and peripheral developing nervous system, and in both neurons as well as glial cells.

Along this line, Sato et al. present a minireview about the estrogen receptors and their close relative the estrogen related receptors (ERRs) that lack estrogen binding capacity (8) in Alzheimer’s disease (AD). These receptors are involved in learning and memory as well as in neuroprotection against neurotoxicity induced by amyloid beta by mechanisms that still remain to be unraveled. Thus, estrogen receptors are not only involved in neurodevelopment and reproduction, but also in healthy brain aging (9). Understanding the role of ERs and ERRs on neurons and glial cells during the aging process could therefore provide new insights into the sex difference in AD and provide new more personalized preventive strategies to combat AD in men and women.

The recently revealed cross-talk of neurotrophic factors with hormones, like glucocorticoids, is reviewed by Tsimpolis et al., presenting the regulation of the major CNS neurotrophin, Brain Derived Neurotrophic Factor (BDNF) functions by glucocorticoids. These pathways are not only crucial for neurogenesis, synaptic plasticity and memory performance, but are also strongly correlated with neuroinflammation, through regulation of microglia and astrocyte function. BDNF and its receptor TrkB activity, as well as glucocorticoid signaling, are highly associated with the onset and progress of most neurological diseases, mainly cognitive disorders and depression (10). Elucidating the tempo-spatial dimension of their interaction could be of great importance towards the understanding of the pathophysiology of brain diseases and the development of innovative therapeutic strategies for cognitive enhancement.

Altogether, there is still much to discover about the complex temporal and spatial actions of steroids on the nervous systems. This field of research is also of major importance for society and public health as these hormones are involved in numerous pathologies of the brain while their synthetic derivatives are also among the most used drugs (11).
